# Safety of Antihypertensive Medication for the Management of Non‐Severe Gestational Hypertension Among Pregnant Individuals in Botswana—Emulating a Series of Target Trials

**DOI:** 10.1111/ppe.70079

**Published:** 2025-10-21

**Authors:** Julia D. DiTosto, Rebecca Zash, Denise L. Jacobson, Katherine Johnson, Modiegi Diseko, Gloria Mayondi, Judith Mabuta, Mompati Mmalane, Joseph Makhema, Sunni L. Mumford, Shahin Lockman, Roger Shapiro, Ellen C. Caniglia

**Affiliations:** ^1^ Department of Biostatistics, Epidemiology, and Informatics, Perelman School of Medicine University of Pennsylvania Philadelphia Pennsylvania USA; ^2^ Botswana‐Harvard AIDS Initiative Partnership for HIV Research and Education Gaborone Botswana; ^3^ Division of Infectious Disease Beth Israel Deaconess Medical Center Boston Massachusetts USA; ^4^ Department of Obstetrics and Gynecology University of Massachusetts Chan Medical School Worcester Massachusetts USA; ^5^ Department of Immunology and Infectious Diseases Harvard T.H. Chan School of Public Health Boston Massachusetts USA; ^6^ Center for Biostatistics in AIDS Research Harvard School of Public Health Boston Massachusetts USA; ^7^ Department of Obstetrics and Gynecology, Perelman School of Medicine University of Pennsylvania Philadelphia Pennsylvania USA; ^8^ Division of Infectious Diseases Brigham and Women's Hospital Boston Massachusetts USA

**Keywords:** antihypertensive medication, gestational hypertension, immortal time bias, target trial emulation

## Abstract

**Background:**

Data on antihypertensive medication for non‐severe gestational hypertension may suffer from immortal time and selection bias. Emulating target trials can prevent these biases by aligning follow‐up with treatment initiation.

**Objectives:**

We estimated the safety of antihypertensive medication initiation for the treatment of non‐severe gestational hypertension on adverse birth outcomes in Botswana using sequential target trial emulation.

**Methods:**

Data from the Tsepamo study (2014–2022), capturing birth outcomes at government delivery sites in Botswana, was used to examine antihypertensive medication initiation ≥ 24 weeks gestation for non‐severe gestational hypertension (140–159 systolic or 90–109 diastolic blood pressure ≥ 20 weeks gestation without chronic hypertension). Sequential weekly target trial emulation compared initiation versus no initiation during 24–35 weeks' gestation on the risk of stillbirth and birth of infant small for gestational age (SGA), with secondary outcomes including very SGA, preterm birth, very preterm birth, neonatal death, and severe gestational hypertension. For each trial, eligible individuals were without chronic hypertension, had not previously initiated antihypertensive medication and had ≥ 2 non‐severe blood pressure readings, at least one within 1 week of trial start. Log‐binomial models estimated gestational week‐specific and pooled risk ratios (RR) with 95% confidence intervals (CI) using bootstrapping.

**Results:**

Of eligible individuals, there were 1676 antihypertensive initiator ‘person‐trials’ and 5211 non‐initiator ‘person‐trials’. In the pooled analysis, the adjusted RR for stillbirth and SGA comparing initiators to non‐initiators was 0.92 (0.68, 1.19) and 1.09 (0.97, 1.23), respectively. The pooled adjusted RR for secondary outcomes were: very SGA, 1.05 (95% CI 0.88, 1.25); preterm birth, 1.09 (95% CI 0.96, 1.22); very preterm birth, 1.05 (95% CI 0.78, 1.47); neonatal death, 1.23 (95% CI 0.68, 2.24); severe gestational hypertension, 0.88 (95% CI 0.74, 1.07).

**Conclusions:**

In this retrospective cohort study, antihypertensive medication initiation between 24 and 35 weeks' gestation for non‐severe gestational hypertension was not associated with increased risk of adverse birth outcomes.

## 1 Introduction

Maternal hypertensive disorders of pregnancy (HDP) affect nearly one in five pregnancies, causing significant maternal and neonatal morbidity and mortality [1, 2]. HDP encompasses chronic hypertension, pre‐eclampsia and related disorders, and gestational hypertension (severe and non‐severe). While antihypertensive treatment for severe HDP is widely accepted [3, 4], the benefits of treating non‐severe gestational hypertension, defined by elevated blood pressures between 140 and 159 systolic or 90–109 diastolic, remain debated.

Antihypertensive medication may prevent progression to severe hypertension, thereby reducing adverse birth outcomes [5]. However, antihypertensive medications raise potential safety concerns, including maternal side effects such as hypotension and reduced uteroplacental perfusion. Additionally, certain agents may cause foetal growth restriction by decreasing uterine blood flow, increasing risks of stillbirth or birth of an infant small‐for‐gestational‐age [6, 7]. In 2018, the World Health Organization (WHO) endorsed treatment for non‐severe gestational hypertension based on a Cochrane review of antihypertensive therapy [8, 9]. However, most trials included in this review also enrolled individuals with severe or chronic hypertension, who are at higher risk of adverse outcomes than those with non‐severe gestational hypertension. Furthermore, most trials were published before 2000, with only two conducted in Sub‐Saharan Africa [10, 11]. Most notably, the Control of Hypertension in Pregnancy Study (CHIPS) found that antihypertensive initiation among individuals with chronic or gestational hypertension reduced adverse outcomes, yet only 25% of the cohort (*N* = 251) had gestational hypertension [5], limiting generalizability to a population exclusively among individuals with non‐severe gestational hypertension. Despite the WHO endorsement, the use of antihypertensive medications for the treatment of non‐severe gestational hypertension is still widely debated, with commonly cited reasons due to limited data exclusively with non‐severe gestational hypertension [12].

Observational studies on antihypertensive use among individuals with non‐severe gestational hypertension, particularly in Sub‐Saharan Africa, are limited and inconsistent [6, 13, 14, 15, 16]. A case–control study in Botswana reported higher odds of antihypertensive use during pregnancy among cases with SGA or stillbirth compared to controls, even after adjusting for important confounders [13]. However, analyses of observational data may be susceptible to immortal time bias [17] by classifying medication use as any time during pregnancy or selection bias by excluding individuals who later developed severe gestational hypertension.

Target trial emulation can reduce these biases by explicitly designing hypothetical trials to better leverage observational data [17]. By explicitly designing a hypothetical trial—the ‘target trial’—researchers can better leverage observational data, reduce bias, and appropriately account for time‐varying exposures. Since non‐severe gestational hypertension can be diagnosed at different gestational ages, a sequence of target trials can be emulated where eligible participants are ‘assigned’ to antihypertensive initiation or no initiation at each gestational week. This study applied these methods to estimate the safety of initiating antihypertensive medication for non‐severe gestational hypertension on adverse birth outcomes in Botswana.

## 2 Methods

### 2.1 Target Trial Framework

We emulated a hypothetical randomised trial—a target trial—to estimate the observational analogue to the intention‐to‐treat effect of antihypertensive initiation for treatment of non‐severe gestational hypertension on adverse birth outcomes [17, 18, 19, 20]. This approach addresses potential biases, including immortal time and selection bias, that commonly affect observational studies. Non‐severe gestational hypertension was defined as systolic blood pressure 140–159 mmHg and/or diastolic blood pressure 90–109 mmHg occurring ≥ 20 weeks' gestation.

### 2.2 Data Source and Study Population

This analysis used data from the Tsepamo study, an ongoing, nationally representative birth outcomes surveillance study in Botswana. The Tsepamo study was created to compare the risk of adverse birth outcomes by maternal antiretroviral therapy among pregnant people living with HIV infection, but pregnant people without HIV are also included [21]. Eight hospitals (45% of all births in Botswana) contributed data to the cohort between 2014 and 2018, and it was expanded to include 18 hospitals (72% of all births) from 2018 to 2022. All pregnant people who delivered live born or stillborn infants after 24 weeks' gestational age at study site government maternity wards in Botswana were included. Importantly, Botswana citizens receive free antenatal care services, HIV testing and medications, including antihypertensive medication. The Tsepamo study has received ethical approval from the Human Research and Development Council in Botswana and by the Institutional Review Board of Harvard T.H. Chan School of Public Health. The need for informed consent was waived for this observational study of deidentified data.

Deidentified data were abstracted from obstetric cards that are used throughout pregnancy at the time of discharge from the postnatal ward. Nurses documented and recorded gestational age based on the last menstrual period at the initial antenatal visit, with confirmation through ultrasonographic assessment [21, 22, 23]. The maternity obstetric record included data on maternal demographics, maternal medical history, HIV status, and self‐reported alcohol and tobacco use. Information also includes repeated blood pressure and weight records, maternal diagnoses, medications prescribed and associated dates, and the infant birth records with the type of delivery, Apgar scores, gestational age, birthweight, congenital abnormalities and vital status of the infants upon discharge.

### 2.3 Outcome Ascertainment

All outcomes were ascertained via obstetric cards. The primary outcomes were the risk of stillbirth and the birth of an SGA infant. Secondary outcomes included the birth of an infant very small for gestational age (VSGA), preterm birth, very preterm birth, in‐hospital neonatal death and the development of severe gestational hypertension.

Stillbirth was defined as Apgar scores of 0 and 0 at 1 and 5 min, respectively; SGA was birth weight below the 10th percentile for gestational age [24], and VSGA was birth weight below the 3rd percentile for gestational age. Preterm birth and very preterm birth, respectively, were defined as birth at a gestational age of < 37 and 32 weeks. Neonatal death included deaths before 28 days after birth among infants who never left the hospital. Development of severe gestational hypertension was defined as a blood pressure reading of ≥ 160 mmHg systolic and/or ≥ 110 mmHg diastolic ≥ 20 weeks' gestation.

### 2.4 Single Target Trial Emulation

We first describe emulating a target trial with enrolment at 24 weeks' gestation and then describe how we can emulate a series of target trials (Table 1) with enrolment from 24 to 35 weeks' gestation. The emulation was stopped at 35 weeks to ensure there would be sufficient biological time for the medication to impact the clinical events of interest [5, 25].

**TABLE 1:** Protocol of target trial and emulation using observational data from the Tsepamo study.

	Target trial protocol	Emulation using Tsepamo study
Eligibility criteria	Currently pregnant with a singleton No late entry (> 20 weeks) to prenatal care No history of chronic hypertension or elevated blood pressure before 20 weeks No prior use of antihypertensive medications At least two elevated non‐severe gestational hypertensive blood pressure reading, one of which must have occurred within 1 week of trial start No prior or current severe blood pressure reading No evidence of proteinuria	Same, except must receive antenatal care at one of the Tsepamo study sites. We do not have data on date of pre‐eclampsia diagnosis, and thus did not know if individuals had pre‐eclampsia at the time of enrolment or after enrolment. We included individuals with pre‐eclampsia diagnosis, assuming that pre‐eclampsia developed after enrolment. This assumption was tested in a sensitivity analysis
Treatment strategies	Initiate antihypertensives at baseline Refrain from taking antihypertensives at baseline	Same
Randomised assignment	Participants will be randomly assigned to either strategy at baseline, and will be aware of the strategy they have been assigned to	Participants are assumed to be randomly assigned at baseline within levels of maternal age, parity, history of preterm or stillbirth, occupation, HIV status, trimester of first antenatal care, first trimester weight, SBP and DBP at non‐severe gestational hypertension diagnosis, SBP and DBP at start of trial, delivery site and calendar year of delivery
Start/end of follow‐up	Followed from baseline (gestational week of trial start) until outcome of interest or discharge from birth hospitalisation	Same
Outcomes	Risk of stillbirth, SGA, VSGA, preterm birth, very preterm birth, neonatal death and development of severe gestational hypertensive blood pressure	Same
Causal contrast of interest	Intention‐to‐treat effect	Observational analogue of the intention‐to‐treat effect, that is, the effect of initiating versus not initiating antihypertensives at baseline
Analysis plan	Intention‐to‐treat analysis	Same, except that estimates are weighted for baseline variables

Abbreviations: SGA, small for gestational age; VSGA, very small for gestational‐age.

### 2.5 Eligibility Criteria

Pregnant individuals were eligible if they: (1) presented to antenatal care before 20 weeks' gestation (any time after is considered late entry to antenatal care in Botswana), (2) had a singleton pregnancy and (3) had at least two elevated, non‐severe blood pressure readings with the first elevated reading occurring at or after 20 weeks' gestation (consistent with the gestational hypertension definition) and one elevated reading within 1 week of 24 weeks' gestation (i.e., between 23 weeks 0 days and 24 weeks 6 days).

### 2.6 Treatment Assignment and Analysis

Eligible individuals were classified as initiators if they started antihypertensive medication between 24 and 25 weeks' gestation and as non‐initiators if they did not start medication during this period. Initiation was defined as the first prescription of methyldopa, nifedipine, or hydralazine after non‐severe gestational hypertension. The primary outcome was the composite of stillbirth or SGA birth. We estimated the observational analogue to the intention‐to‐treat effect using a log‐binomial regression model to calculate risk ratios.

### 2.7 Baseline Confounding Adjustment

To emulate randomization, we used stabilised inverse probability of treatment weighting (IPTW) to adjust for baseline confounding. The treatment model included: maternal age (< 18, 18–35, ≥ 35 years), parity (nulliparous vs. parous), history of preterm birth or stillbirth, occupation (salaried vs. other/unknown), HIV status, trimester of first antenatal care visit (first [< 12 weeks] vs. second [12–20 weeks]), first trimester weight (< 50, 50–80, ≥ 80 kg) and systolic and diastolic blood pressure values at the first non‐severe gestational hypertensive reading and at the qualifying reading within 1 week of 24 weeks. Unknown or missing was rare (< 5%) and collapsed into the most prevalent category. Blood pressures were modelled using restricted cubic splines with three knots. We also included delivery site (urban vs. rural) and calendar year of delivery (2014–2016, 2017–2019, 2020–2022) to account for geographic and temporal variability.

### 2.8 Sequential Target Trial Emulation

We emulated a series of 12 target trials, each with 1‐week enrolment periods from 24 to 35 weeks' gestation. The same eligibility criteria were applied at each gestational week, with the timing requirement adjusted accordingly. For example, the trial at 25 weeks enroled individuals with their second non‐severe elevated blood pressure reading at 25 weeks who had not yet initiated antihypertensive medication.

An individual could be eligible for multiple sequential trials. If an individual was eligible but did not initiate treatment in a given week, they were classified as a non‐initiator for that trial. In the week they initiated treatment, they were classified as an initiator for that trial. This approach allows individuals to contribute data as non‐initiators in early trials and as initiators in later trials, reflecting real‐world treatment patterns while avoiding immortal time bias [17].

For the outcome of very preterm birth (birth < 32 weeks), only seven trials were emulated (24–30 weeks) since the outcome must occur before 32 weeks.

### 2.9 Statistical Analysis

For sequential trial emulation, baseline covariates were updated at the start of each trial using the most recent measurements available at that gestational age. Data from all trials were pooled into a single weighted log‐binomial regression model that included ‘trial week’ (values 1–12) as a covariate to account for the different enrolment periods.

All analyses were performed in SAS 9.4. Confidence intervals were obtained using bootstrapping with 200 samples, chosen to balance computational efficiency with precision. Figures were created using RStudio.

### 2.10 Sensitivity Analyses

We conducted four sensitivity analyses to assess the robustness of our findings. First, to ensure the two elevated blood pressure readings occurred close together in time, we (1) required both readings to occur within 1 week of each other rather than our original broader timeframe to mimic an RCT where more than one blood pressure measurement would be made to determine eligibility. To minimise potential residual immortal time bias from our 1‐week treatment initiation window, we (2) required antihypertensive initiation on the same day as the second elevated blood pressure reading. Third, since the Tsepamo study lacks data on pre‐eclampsia diagnosis timing, we (3) excluded all individuals ever diagnosed with pre‐eclampsia to ensure we did not inadvertently include those diagnosed prior to trial start. Finally, we (4) revised the eligibility assessment to occur at the end of the trial week to align with when treatment ascertainment was evaluated. Figure S1 displays a schematic for these analytic variations.

### 2.11 Subgroup Analyses

We explore effect modification by parity, HIV status and first trimester weight in subgroup analyses restricted to (1) nulliparous; (2) primiparous; (3) individuals living with HIV; (4) individuals not living with HIV; (5) < 5 0 kg first trimester weight; and (6) ≥ 80 kg first trimester weight.

### 2.12 Missing Data

Individuals were excluded if their antihypertensive start date (*N* = 14, < 0.001%) or age (*N* = 162, < 0.01%) was missing. There were no missing data for the outcomes. Missing data for covariates were under 5%, and we collapsed missingness with the most prevalent condition.

## 3 Results

Of 23,436 pregnant individuals eligible for any target trial, 1676 (7.1%) initiated antihypertensive medication at some point between 24 and 35 weeks' 6 days (Figure S2). This corresponds to 1676 initiator ‘person‐trials’ and 5211 non‐initiator ‘person‐trials’ (Table 2). The percentage of initiators increased throughout pregnancy: 4.2% (52/176) initiated antihypertensive medication in Week 24 compared to 11.8% (103/978) in Week 35 (3–4).

**TABLE 2:** Baseline characteristics of antihypertensive initiators and non‐initiators between 24 and 35 weeks gestation, the Tsepamo study (2014–2022).

	Overall (6887 person‐trials)	AH initiators (1676 person‐trials)	AH non‐initiators (5211 person‐trials)
Maternal age, years, *N* (%)			
< 18 years	2382 (34.6)	565 (33.7)	1817 (34.9)
18–35 years	2977 (43.2)	741 (44.2)	2236 (42.9)
≥ 35 years	1528 (22.2)	370 (22.1)	1158 (22.2)
Nulliparous, *N* (%)	2824 (41.0)	672 (40.1)	2152 (41.3)
History of preterm birth or stillbirth, *N* (%)			
Yes	469 (6.8)	125 (7.5)	344 (6.6)
No	3755 (54.5)	922 (55.0)	2833 (54.4)
Unknown or first pregnancy	2663 (38.7)	629 (37.5)	2034 (39.0)
Salaried occupation, *N* (%)	2578 (37.4)	639 (38.1)	1939 (37.2)
Living with HIV, *N* (%)	1422 (20.6)	328 (19.6)	1094 (21.0)
First trimester initiation of antenatal care, *N* (%)	1382 (20.1)	348 (20.8)	1034 (19.8)
Rural site, *N* (%)	3200 (46.5)	913 (54.5)	2287 (43.9)
First trimester weight, *N* (%)			
< 50 kg	484 (7.0)	131 (7.8)	353 (6.8)
50–80 kg	4283 (62.2)	1072 (64.0)	3211 (61.6)
≥ 80 kg	2120 (30.8)	473 (28.2)	1647 (31.6)
Calendar year of delivery, *N* (%)			
2014–2016	1919 (27.9)	496 (29.6)	1423 (27.3)
2017–2019	2351 (34.1)	579 (34.6)	1772 (34.0)
2020–2022	2617 (38.0)	601 (35.9)	2016 (38.7)
SBP/DBP at diagnosis, median (IQR)	141 (134–146)/90 (81–93)	141 (134–146)/90 (84–93.5)	141 (133–146)/90 (80–93)
SBP/DBP at trial start, median (IQR)	142 (135–147)/91 (84–94)	144 (140–149)/93 (88–97)	141 (133–146)/90 (82–93)

Abbreviations: DBP, diastolic blood pressure; IQR, interquartile range; SBP, systolic blood pressure.

Initiators and non‐initiators had similar baseline characteristics, except that initiators were more likely to receive antenatal care at a rural site and were less likely to deliver between 2020 and 2022 (Table 2). Nearly all initiators (94.6%, *N* = 1585) were prescribed methyldopa (Table 3). Less commonly initiated medications were nifedipine and hydralazine.

**TABLE 3:** Frequency of initiating each type of antihypertensive between 24 and 25 weeks' gestation and timing of antihypertensive initiation, the Tsepamo study (2014–2022).

Type of antihypertensive	Number (%^a^)	Median (IQR) weeks at initiation
Methyldopa	1585 (94.6)	32 (30–34)
Nifedipine	118 (7.0)	32 (30–34)
Hydralazine	3 (0.2)	32 (27–35)

Abbreviation: IQR, interquartile range.

^a^ Percentages sum to more than 100% because individuals could have initiated more than one type of antihypertensive on the same date.

**FIGURE 1:**
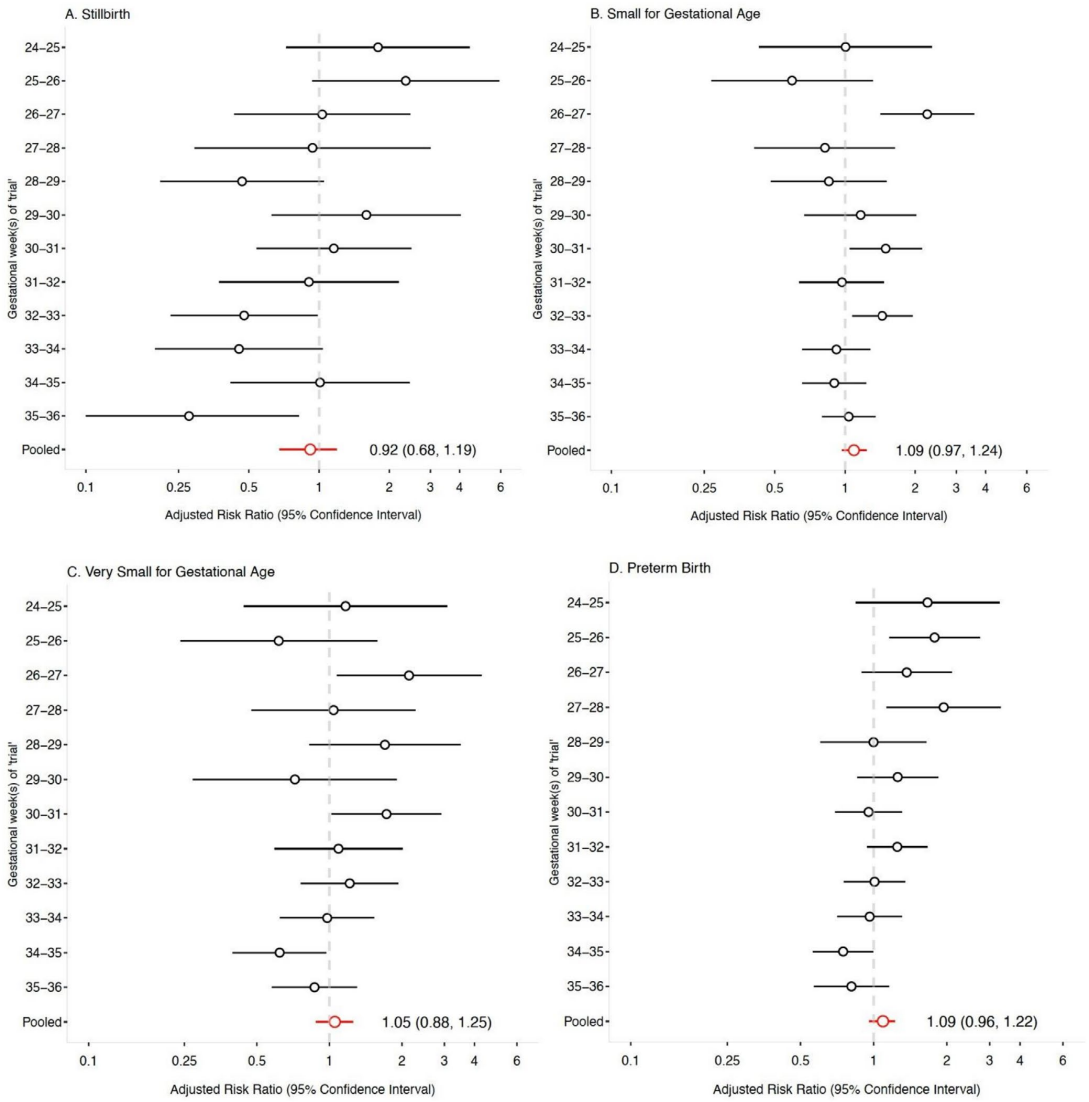
Adjusted risk ratios and 95% confidence intervals for primary and exploratory outcomes comparing antihypertensive initiation to no initiation between 24 and 35 weeks' gestation, the Tsepamo study (2014–2022). Models were adjusted using stabilised inverse probability weights accounting for maternal age, parity, history of preterm or stillbirth, occupation, HIV status, trimester of first antenatal care, first trimester weight, first SBP and DBP at non‐severe gestational hypertension diagnosis, SBP and DBP at start of trial, delivery site and calendar year of delivery. The adjusted pooled model additionally includes ‘trial’ as an adjustment variable. 95% CI for the pooled models were calculated via bootstrapping with 200 samples. (A) Stillbirth; (B) Small for Gestational Age; (C) Very Small for Gestational Age; (D) Preterm birth.

Among initiators, 6.6% (*N* = 111) of pregnancies resulted in a stillbirth compared to 5.2% (*N* = 269) among the non‐initiators across all trials (Table S1). The adjusted RRs for stillbirth ranged from 0.19 (95% CI 0.02, 1.49) to 2.35 (95% CI 0.94, 5.91) (Figure 1A). In the pooled analysis, the adjusted RR for stillbirth comparing initiators to non‐initiators was 0.92 (95% CI 0.68, 1.19). Among initiators, 27.9% (*N* = 467) of infants were born SGA compared to 21.9% (*N* = 1141) among the non‐initiators across all trials (Table S1). The adjusted RRs for SGA ranged from 0.59 (95% CI 0.27, 1.31) to 2.25 (95% CI 1.42, 3.57) (Figure 1B). In the pooled analysis, the adjusted RR for SGA comparing initiators to non‐initiators was 1.09 (95% CI 0.97, 1.23).

**FIGURE 2:**
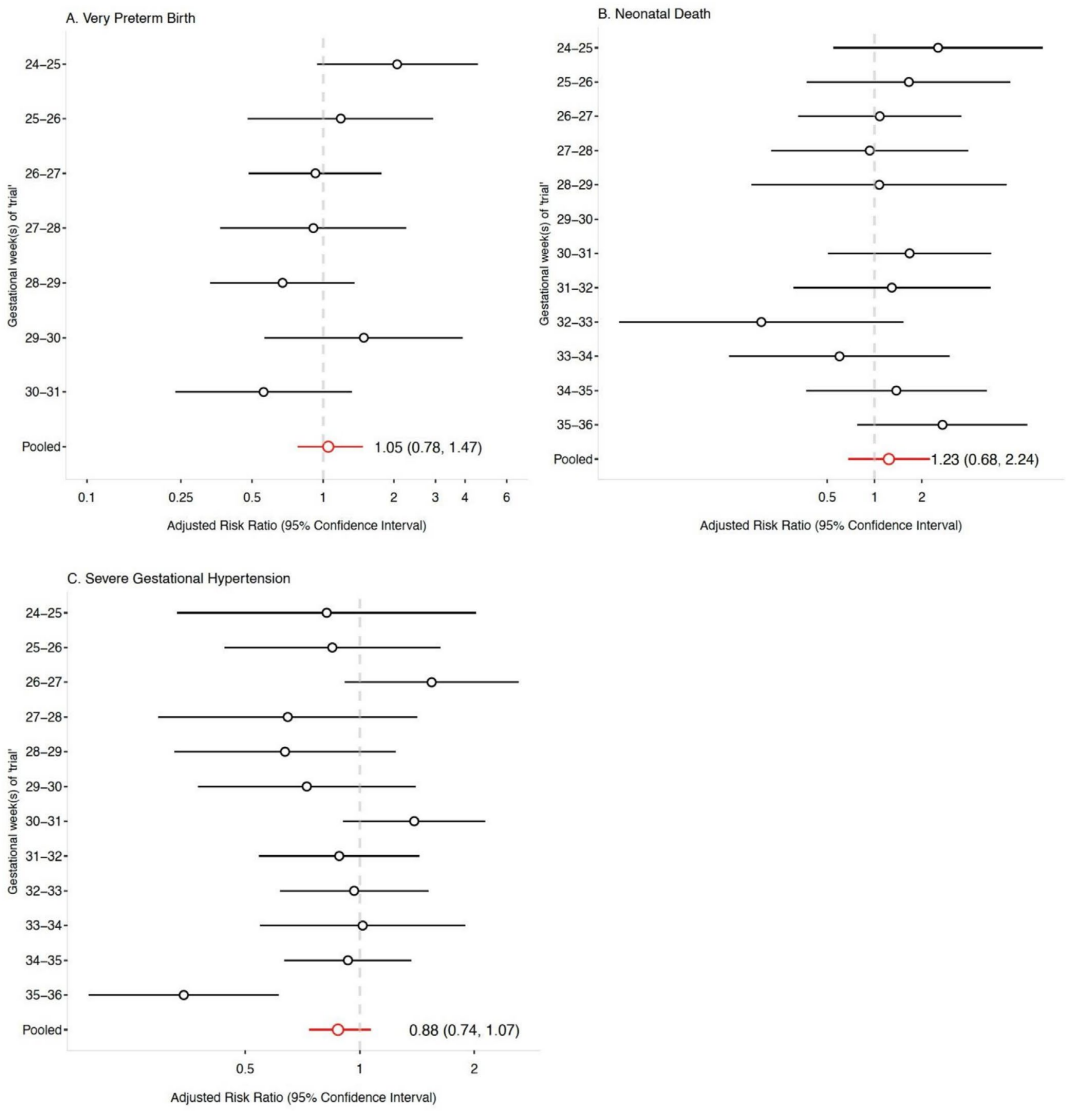
Adjusted risk ratios and 95% confidence intervals for exploratory outcomes comparing antihypertensive initiation to no initiation between 24 and 35 weeks' gestation, the Tsepamo study (2014–2022). Models were adjusted using stabilised inverse probability weights accounting for maternal age, parity, history of preterm or stillbirth, occupation, HIV status, trimester of first antenatal care, first trimester weight, first SBP and DBP at non‐severe gestational hypertension diagnosis, SBP and DBP at start of trial, delivery site and calendar year of delivery. The adjusted pooled model additionally includes ‘trial’ as an adjustment variable. 95% CI for the pooled models were calculated via bootstrapping with 200 samples. (A) Very Preterm Birth; (B) Neonatal Death; (C) Severe Gestational Hypertension.

We examined the risk of birth to an infant VSGA, preterm birth, very preterm birth, neonatal death and development of severe gestational hypertension as secondary outcomes in initiators compared to non‐initiators (Figure 1C,D; Figure 2; Table S1). The pooled adjusted RR for each secondary outcome was: VSGA, 1.05 (95% CI 0.88, 1.25); preterm birth, 1.09 (95% CI 0.96, 1.22); very preterm birth, 1.05 (95% CI 0.78, 1.47); neonatal death, 1.23 (95% CI 0.68, 2.24); severe gestational hypertension, 0.88 (95% CI 0.74, 1.07).

The aRR for stillbirth was 0.65 (95% CI 0.46, 1.17) in the sensitivity analysis evaluating initiation of antihypertensive medication the week of the first elevated blood pressure, 0.82 (95% CI 0.58, 1.11) in the sensitivity analysis evaluating initiation of antihypertensive medication the day of the elevated blood pressure, and 0.61 (95% CI 0.38, 1.11) in the sensitivity analysis excluding those ever diagnosed with pre‐eclampsia, and 0.99 (95% CI 0.75, 1.34) in the sensitivity analysis assessing eligibility criteria at the end of the week (Table 4), though confidence intervals were wide. Risk ratios for SGA were similar to the primary analysis across the sensitivity analyses. Risk ratios for secondary outcomes were generally similar in sensitivity analyses compared with the primary analyses. However, the aRR for very preterm birth was 0.66 (95% CI 0.38, 1.02) in the sensitivity analysis excluding those ever diagnosed with pre‐eclampsia, compared to the primary analysis of 1.05 (95% CI 0.78, 1.47).

**TABLE 4:** Primary and sensitivity analyses of risk ratios of stillbirth by antihypertensive initiation between 24 and 35 weeks' gestation, the Tsepamo study (2014–2022).

	Primary analysis	S#1: Require elevated blood pressures to be within 1 week	S#2: Must initiate antihypertensives on the same day of elevated blood pressure	S#3: Exclude those who were ever diagnosed with pre‐eclampsia	S#4: Assess eligibility at the end of the trial enrolment week
Outcome: stillbirth					
No events/Total no person‐trials (%) Non‐initiators	269/5211 (5.2)	67/821 (8.2)	278/5265 (5.3)	218/4500 (4.8)	231/5130 (4.5)
No events/Total no person‐trials (%) Initiators	111/1474 (7.5)	46/617 (7.5)	102/1621 (6.3)	49/1056 (4.6)	102/1610 (6.3)
Pooled aRR^a^ (95% CI)^b^	0.92 (0.68, 1.19)	0.65 (0.46, 1.17)	0.82 (0.58, 1.11)	0.61 (0.38, 1.02)	0.99 (0.75, 1.34)
Outcome: SGA					
No events/Total no person‐trials (%) Non‐initiators	1141/5175 (22.0)	235/816 (28.8)	1159/5229 (22.2)	885/4466 (19.8)	1116/5094 (21.9)
No events/Total no person‐trials (%) Initiators	467/1665 (28.0)	194/613 (31.6)	449/1611 (27.9)	246/1052 (23.4)	447/1600 (27.9)
Pooled aRR^a^ (95% CI)^b^	1.09 (0.97, 1.23)	0.97 (0.80, 1.20)	1.07 (0.95, 1.23)	1.10 (0.94, 1.29)	1.08 (0.96, 1.25)
Outcome: VSGA					
No events/Total no person‐trials (%) Non‐initiators	554/5175 (10.7)	122/816 (15.0)	565/5229 (10.8)	396/4466 (8.9)	542/5094 (10.6)
No events/Total no person‐trials (%) Initiators	249/1665 (15.0)	97/613 (15.8)	238/1611 (14.8)	114/1052 (10.8)	237/1600 (14.8)
Pooled aRR^a^ (95% CI)^b^	1.05 (0.88, 1.25)	0.91 (0.61, 1.28)	1.02 (0.86, 1.23)	1.00 (0.75, 1.32)	1.04 (0.88, 1.22)
Outcome: preterm birth					
No events/Total no person‐trials (%) Non‐initiators	1337/5211 (25.7)	346/821 (42.1)	1366/5265 (25.9)	1035/4500 (23.0)	1207/5130 (23.5)
No events/Total no person‐trials (%) Initiators	606/1474 (41.1)	284/617 (46.0)	577/1622 (35.6)	308/1056 (29.2)	566/1611 (35.1)
Pooled aRR^a^ (95% CI)^b^	1.09 (0.96, 1.22)	0.94 (0.80, 1.07)	1.04 (0.91, 1.17)	0.97 (0.82, 1.14)	1.13 (0.99, 1.34)
Outcome: very preterm birth					
No events/Total no person‐trials (%) Non‐initiators	181/1918 (9.4)	28/751 (3.7)	185/1944 (9.5)	165/2170 (7.6)	149/1938 (7.7)
No events/Total no person‐trials (%) Initiators	97/595 (16.3)	18/568 (3.2)	93/569 (16.3)	39/400 (9.8)	92/568 (16.2)
Pooled aRR^a^ (95% CI)^b^	1.05 (0.78, 1.47)	1.14 (0.55, 1.63)	0.97 (0.71, 1.37)	0.66 (0.38, 1.02)	1.19 (0.88, 1.71)
Outcome: neonatal death					
No events/Total no person‐trials (%) Non‐initiators	81/4938 (1.6)	65/278 (23.4)	84/4983 (1.7)	58/4283 (1.4)	80/4896 (1.6)
No events/Total no person‐trials (%) Initiators	39/1541 (2.5)	57/233 (24.5)	36/1514 (2.4)	21/1005 (2.1)	36/1503 (2.4)
Pooled aRR^a^ (95% CI)^b^	1.23 (0.68, 2.24)	0.93 (0.28, 1.88)	1.12 (0.68, 2.00)	1.23 (0.65, 2.48)	1.16 (0.55, 1.74)
Outcome: severe gestational hypertension					
No events/Total no person‐trials (%) Non‐initiators	743/5211 (14.2)	122/821 (14.9)	755/5265 (14.3)	510/4500 (11.3)	766/5130 (14.9)
No events/Total no person‐trials (%) Initiators	275/1676 (16.4)	113/617 (18.3)	263/1622 (16.2)	140/1056 (13.3)	263/1611 (16.3)
Pooled aRR^a^ (95% CI)^b^	0.88 (0.74, 1.07)	1.21 (0.86, 1.63)	0.84 (0.70, 1.04)	0.85 (0.67, 1.08)	0.80 (0.68, 0.97)

^a^ Models were adjusted using stabilised inverse probability weights accounting for maternal age, parity, history of preterm or stillbirth, occupation, HIV status, trimester of first antenatal care, first trimester weight, first SBP and DBP at non‐severe gestational hypertension diagnosis, SBP and DBP at start of trial, delivery site and calendar year of delivery. The adjusted pooled model additionally includes ‘trial’ as an adjustment variable.

^b^ 95% CI for the pooled models was calculated via bootstrapping with 200 samples.

Sample sizes for subgroup analyses were limited, and results should be interpreted as exploratory (Table S2). The risk ratio for stillbirth comparing antihypertensive initiation with no initiation was lower than the primary analysis among those living with HIV (0.64, 95% CI 0.43, 1.03), < 50 kg first trimester weight (0.54, 95% CI 0.28, 0.97) and ≥ 80 kg first trimester weight (0.65, 95% CI 0.42, 1.01). The risk ratio for SGA was higher than the primary analysis among those < 50 kg first trimester weight (1.57, 95% CI 1.10, 2.02). The results were consistent across parity subgroups.

## 4 Comment

### 4.1 Principal Findings

This study applied a series of emulated target trials to assess the safety of initiating antihypertensive medication for non‐severe gestational hypertension between 24 and 35 weeks' gestation. Overall, antihypertensive initiation was not associated with the risk of stillbirth and SGA. While there was a trend towards increased risk of SGA, the wide confidence intervals suggest limited precision, and confounding by indication cannot be ruled out. Risks of the secondary outcomes of VSGA, preterm birth, very preterm birth, neonatal death and severe gestational hypertension were similar after initiating antihypertensive medication compared to non‐initiation.

### 4.2 Strengths of the Study

An important strength of this study was the use of a large, representative cohort of pregnant individuals who received care at government facilities in Botswana. Our methodological approach was strengthened by implementing sequential target trial methodology, which allowed us to leverage time‐varying clinical data collected throughout pregnancy, including repeated blood pressure measurements. This analytical framework provided clear definitions of the research question and treatment, while simultaneously reducing common biases that typically affect observational research, particularly immortal time bias and selection bias.

### 4.3 Limitations of the Data

Our study has several limitations. First, we lacked data on prescribing practices. In Botswana, clinicians may delay initiating antihypertensive medication for pregnant individuals with non‐severe elevated blood pressure, opting to wait for subsequent high readings, which potentially delays intervention as prenatal visits become more frequent later in pregnancy. This may explain the increase in initiators with progressing gestational weeks. Second, though we included a wide range of time‐varying covariates, residual confounding may prevent a causal interpretation of our findings, as there may have been unmeasured clinical characteristics related to decisions on antihypertensive initiation and risk of adverse birth outcomes. Third, examining the prevention of severe HDP would have been of great clinical interest, but we lacked data on the timing of preeclampsia diagnoses. To address this, we included the development of severe blood pressures as a secondary outcome and conducted sensitivity analyses excluding individuals with a preeclampsia diagnosis at any time point, which did not appreciably change the results. Fourth, the generalizability of our findings to other global populations may be limited due to the distinctive characteristics of our Botswana cohort, including higher incidence rates of the study outcomes relative to other countries, elevated baseline HIV prevalence, and predominant use of methyldopa rather than other antihypertensive agents more commonly prescribed elsewhere.

### 4.4 Interpretation

Existing data in this area remain limited. A meta‐analysis of randomised trials on antihypertensive medication for non‐severe pregnancy hypertension found no significant association between treatment initiation and adverse birth outcomes [26]. However, these results were stratified by drug type, confidence intervals were wide, and stillbirth was not included as an outcome. Notably, antihypertensive initiation was associated with a 30%–70% reduction in the risk of developing severe‐range blood pressures, substantially higher than the 12% reduction observed in our study (aRR 0.88 [95% CI 0.74, 1.07]). Interestingly, the meta‐analysis included studies regardless of the type of pregnancy hypertension, allowing trials to enrol individuals with chronic hypertension if their blood pressures were in the non‐severe range. Additionally, it did not exclude participants with prior antihypertensive medication use, potentially introducing bias, as individuals with prior use across pregnancies may have experienced better outcomes due to earlier successful treatment.

Though specific research on non‐severe gestational hypertension is sparse, broader evidence on mild to moderate HDP shows consistent findings. Both a Cochrane review and meta‐analysis concluded that antihypertensive treatment effectively prevents progression to severe hypertension but demonstrates no clear benefit on maternal or neonatal outcomes [27, 28], aligning with our result and suggesting the primary benefit may be limited to blood pressure control. Notably, the meta‐analysis included various HDP and did not exclude those with prior antihypertensive use, potentially introducing bias as individuals with prior use across pregnancies may have experienced better outcomes due to earlier successful treatment.

To our knowledge, only one other observational study has examined the impact of antihypertensive medication use on adverse birth and maternal outcomes among those with non‐severe gestational hypertension in Botswana. A nested case–control study using Tsepamo data reported that individuals with non‐severe gestational hypertension who experienced SGA or stillbirth had 28% and 42% higher odds, respectively, of using antihypertensive medication during pregnancy compared to controls [13]. Antihypertensive use was defined as use at any time after the diagnosis of gestational hypertension, potentially introducing immortal time bias, as treatment initiation may have occurred after follow‐up began. Immortal time bias typically causes bias away from the null, which would make the exposure of interest harmful; this bias has recently been demonstrated in an example of antibiotics and preterm birth [17]. Additionally, the study excluded individuals who developed severe gestational hypertension, potentially introducing selection bias. In both of these cases, bias could skew results away from the null [17, 29], which may explain the discrepancies in study results.

The proposed mechanism by which antihypertensive medication improves birth outcomes in individuals with non‐severe gestational hypertension is by reducing the risk of developing severe‐range blood pressures. Our findings, showing only a 12% reduced risk (95% CI: 26% decrease to 7% increase) in the development of severe gestational hypertension, suggest that it is unsurprising that associations with other outcomes were modest. This may reflect residual confounding, particularly confounding by indication. While we employed rigorous methods to emulate target trials, residual confounding remains a limitation in observational analyses. Specifically, more severe clinical presentations among those who initiated antihypertensives may have gone unmeasured in the Tsepamo study and could not be fully adjusted for. However, we were able to account for several critical confounders, including systolic and diastolic blood pressure measured at multiple time points, to estimate the effects of antihypertensive use on adverse birth outcomes.

Another potential explanation for why we did not see a robust protective effect of antihypertensive initiation on the risk of adverse birth outcomes could be residual immortal time bias. In this analysis, we defined the target trial emulations on the gestational week scale, meaning that eligible individuals had the entire week to enrol in the hypothetical trial. However, defining trials on the day scale would have been infeasible given the sample size and frequency of treatment initiation. We tested the robustness of this assumption in sensitivity analyses, requiring stricter criteria and shorter time periods for enrolment, and our results were not appreciably different, though confidence intervals were wide. Since we were unable to distinguish between iatrogenic and spontaneous preterm birth, we included very preterm birth as a secondary outcome as most births prior to 32 weeks are spontaneous [30]. There may also be differential effects on outcomes by antihypertensive type; in this cohort, methyldopa was the predominant medication prescribed, which may be less effective at preventing severe hypertension than other agents [9].

## 5 Conclusion

In conclusion, we found little evidence that antihypertensive treatment for non‐severe gestational hypertension between 24 and 35 weeks affects adverse birth outcomes. Further research is needed to better understand the impact of treatment timing on pregnancy outcomes.

## Author Contributions

J.D.D. executed the methodology, carried out the final analysis, and drafted the manuscript. E.C.C. proposed the study and critically reviewed and revised the manuscript. All coauthors supervised the analysis and critically reviewed and revised the manuscript. The final version of the manuscript was approved by all authors.

## Conflicts of Interest

The authors declare no conflicts of interest.

## Supporting information


**Data S1:** ppe70079‐sup‐0001‐supinfo.docx.

## Data Availability

Data and code are available upon reasonable request and approval from the necessary Institutional Review Boards in the United States and Botswana.
