# Supplementary material for: Safety of Antihypertensive Medication for the Management of Non‐Severe Gestational Hypertension Among Pregnant Individuals in Botswana—Emulating a Series of Target Trials
Source: Paediatr Perinat Epidemiol. 2025 Oct 21;40(2):248–60. doi: 10.1111/ppe.70079 (PMC13010225; doi:10.1111/ppe.70079)
Supplement: Supplementary file 1 — Data S1: ppe70079‐sup‐0001‐supinfo.docx. [file PPE-40-248-s001.docx]

Supplemental Material, DiTosto et al.

[Supplemental Figure 1. Schematic of eligibility criteria assessment and treatment ascertainment for the primary and sensitivity analyses. 2](#_Toc200982058)

[Supplemental Figure 2. Flowchart of selection criteria for individuals eligible for any sequential target trial on estimating the safety of antihypertensives for non-severe gestational hypertension, the Tsepamo Study. 3](#_Toc200982059)

[Supplemental Figure 3. Flowchart of target trials of antihypertensive initiation at each week, 24-29 weeks’ gestation. 4](#_Toc200982060)

[Supplemental Figure 4. Flowchart of target trials of antihypertensive initiation at each week, 30-35 weeks’ gestation. 5](#_Toc200982061)

[Supplemental Table 1. Risk ratio of outcomes by antihypertensive initiation between 24- and 35-weeks’ gestation, the Tsepamo Study (2014-2022). 6](#_Toc200982062)

[Supplemental Table 2. Risk ratios from subgroup analyses by HIV status, first trimester weight, and parity on stillbirth and SGA by antihypertensive initiation between 24- and 35-weeks’ gestation, the Tsepamo Study (2014-2022). 9](#_Toc200982063)

# Figure Sx1. Schematic of eligibility criteria assessment and treatment ascertainment for the primary and sensitivity analyses.

**
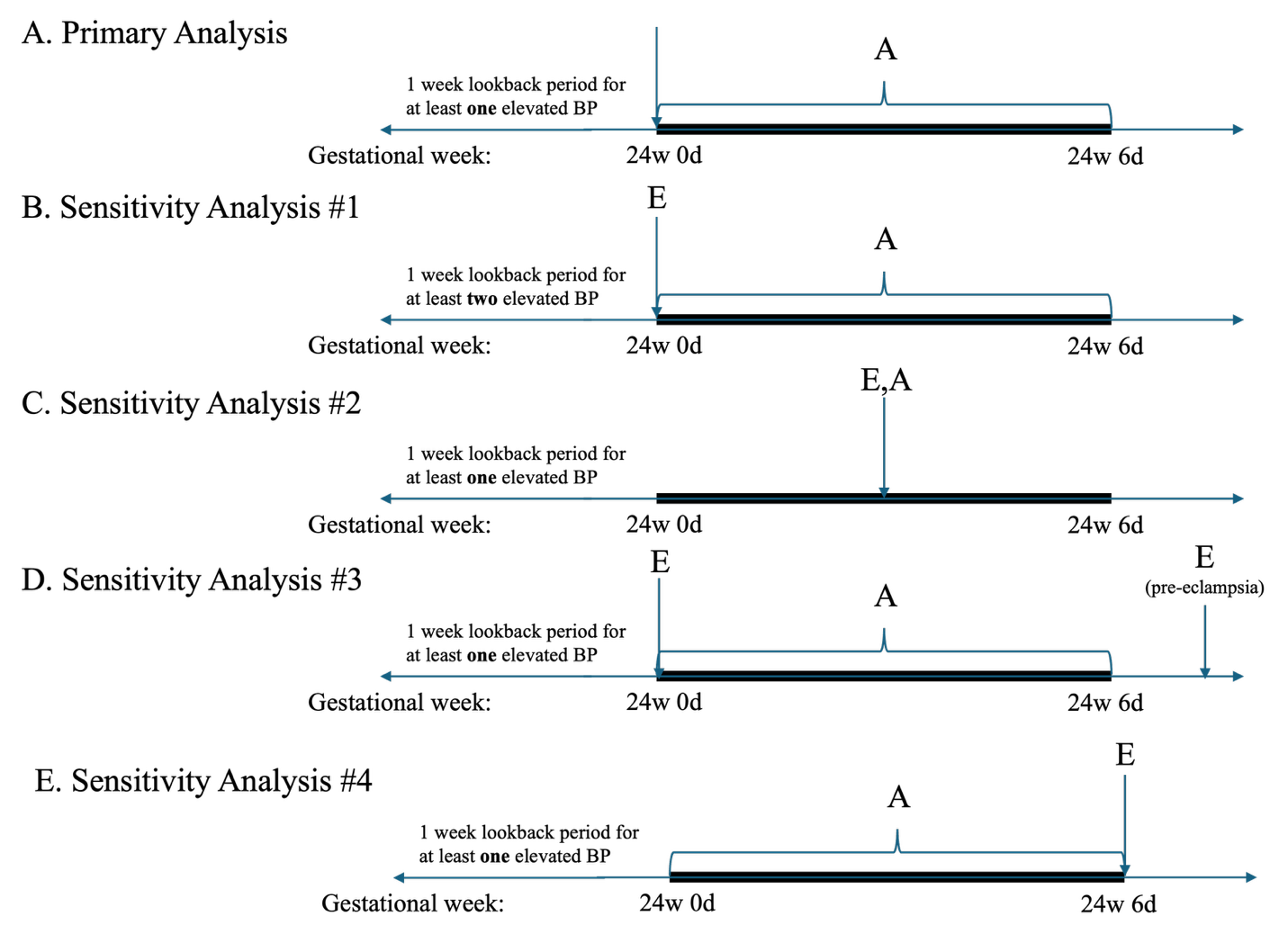
**

A: treatment assignment; E: assessment of eligibility criteria

# Figure Sx2. Flowchart of selection criteria for individuals eligible for any sequential target trial on estimating the safety of antihypertensives for non-severe gestational hypertension, the Tsepamo Study.


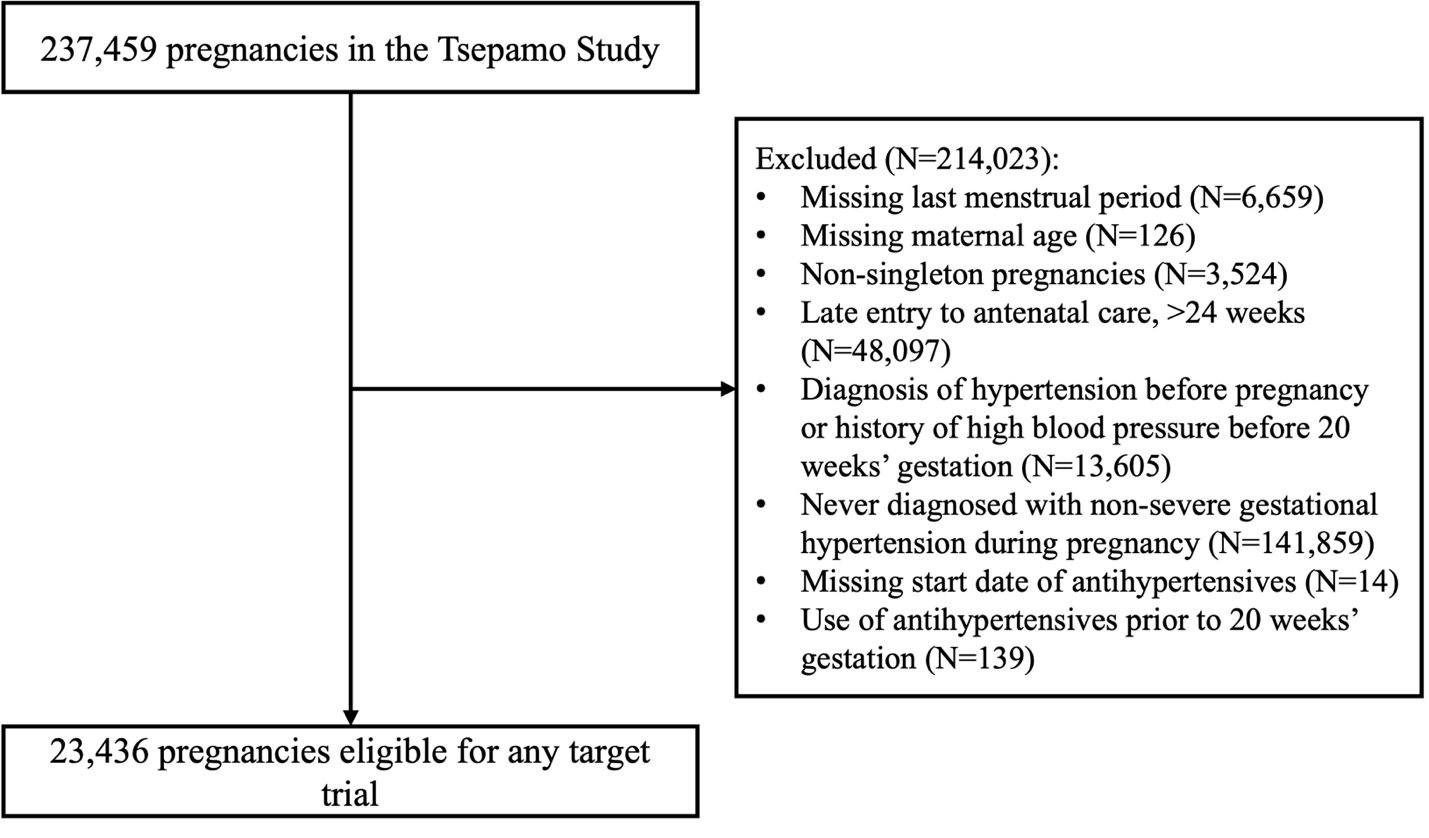


# Figure Sx3. Flowchart of target trials of antihypertensive initiation at each week, 24-29 weeks’ gestation.


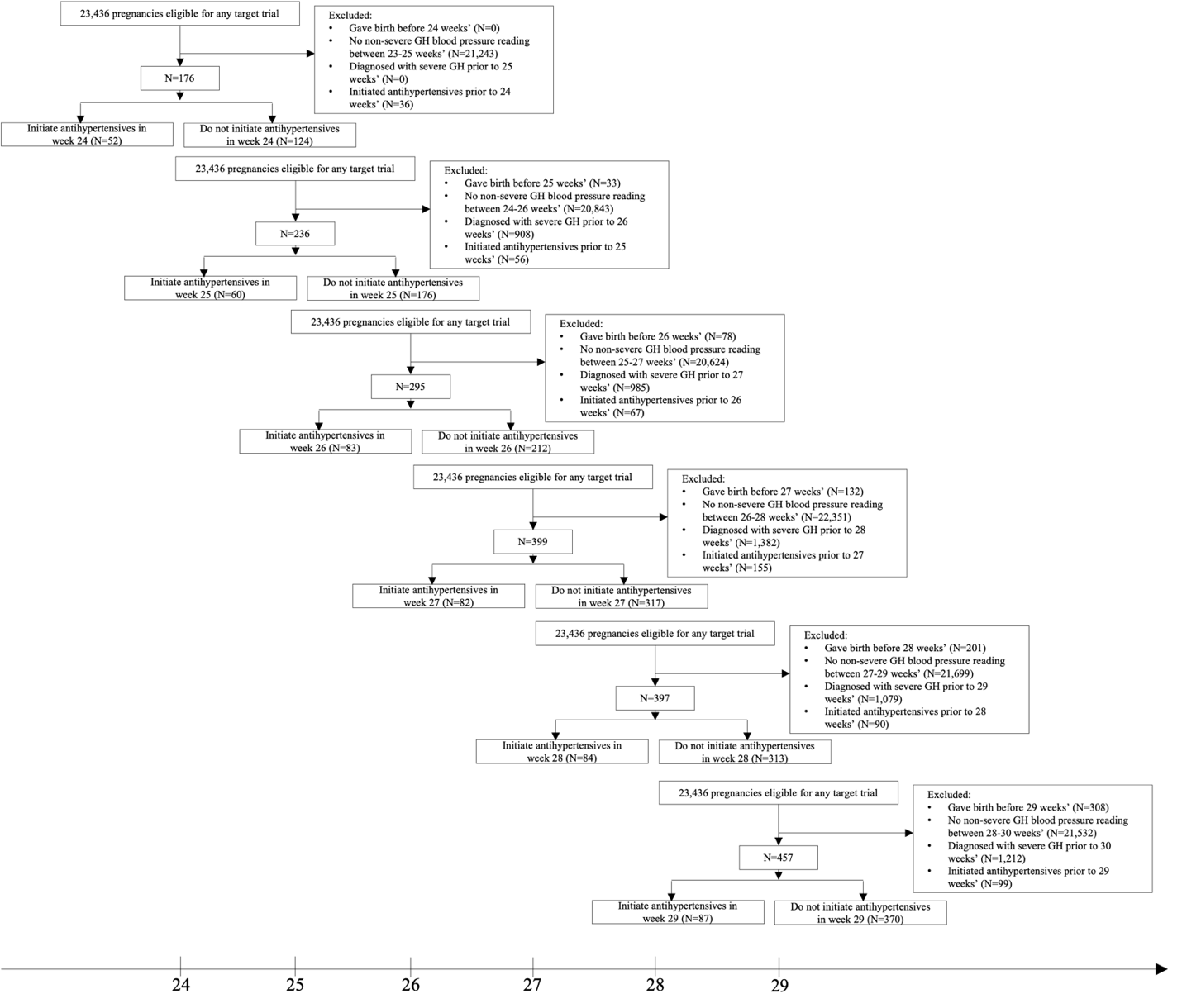


# Figure Sx4. Flowchart of target trials of antihypertensive initiation at each week, 30-35 weeks’ gestation.


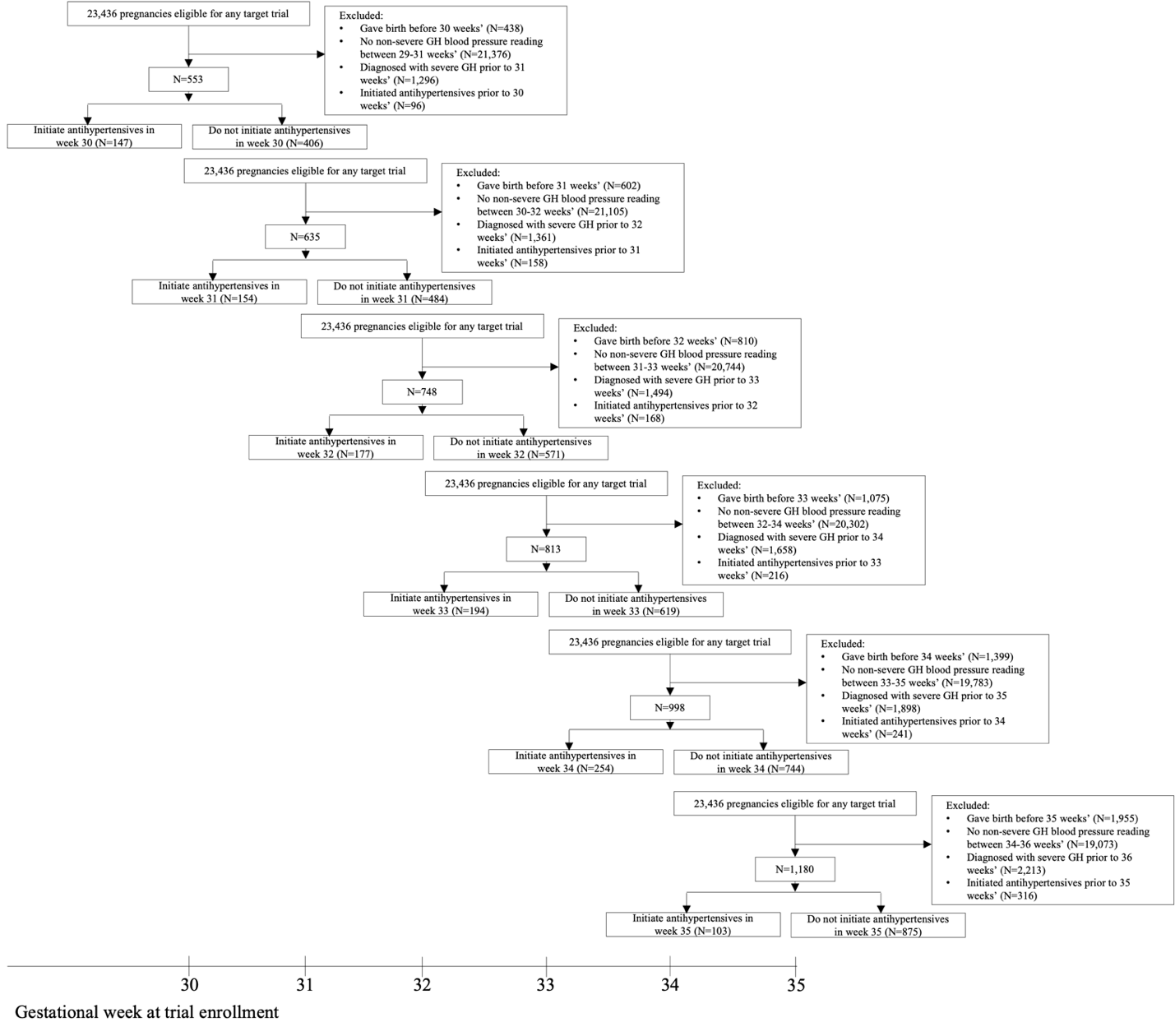


# Table Sx1. Risk ratio of outcomes by antihypertensive initiation between 24- and 35-weeks’ gestation, the Tsepamo Study (2014-2022).

|  | **Outcome: Stillbirth** | | | | | | | | | **Outcome: SGA** | | | | | | | | |  |
| --- | --- | --- | --- | --- | --- | --- | --- | --- | --- | --- | --- | --- | --- | --- | --- | --- | --- | --- | --- |
|  | **Non-initiator** | | **Initiator** | |  | |  | | | | **Non-initiator** | | | **Initiator** | |  |  | | |
| **Trial GA week** | **No events / Total no (%)** | | **No events / Total no (%)** | | **RR (95% CI)** | | **aRR^a^ (95% CI)^b^** | | | | **No events / Total no (%)** | | | **No events / Total no (%)** | | **RR (95% CI)** | **aRR^a^ (95% CI)^b^** | | |
| 24-25 | 17/124  (13.7) | | 12/52  (23.1) | | 1.68  (0.87, 3.27) | | 1.79  (0.72, 4.42) | | | | 25/124  (20.2) | | | 12/51  (23.5) | | 1.17  (0.64, 2.14) | 1.00  (0.43, 2.35) | | |
| 25-26 | 14/176  (8.0) | | 10/60  (16.7) | | 2.10  (0.98, 4.47) | | 2.35  (0.94, 5.91) | | | | 34/175  (19.4) | | | 8/59  (13.6) | | 0.70  (0.34, 1.42) | 0.59  (0.27, 1.31) | | |
| 26-27 | 14/212  (6.6) | | 11/83  (13.2) | | 2.01  (0.95, 4.24) | | 1.03  (0.43, 2.46) | | | | 36/210  (17.1) | | | 29/82  (35.4) | | 2.06  (1.36, 3.13) | 2.25  (1.42, 3.57) | | |
| 27-28 | 22/317  (6.9) | | 8/82  (9.8) | | 1.41  (0.65, 3.04) | | 0.94  (0.29, 3.00) | | | | 57/313  (18.2) | | | 22/80  (27.5) | | 1.51  (0.99, 2.31) | 0.82  (0.41, 1.64) | | |
| 28-29 | 24/313  (7.7) | | 10/84  (11.9) | | 1.55  (0.77, 3.12) | | 0.47  (0.21, 1.05) | | | | 64/310  (20.7) | | | 25/84  (29.8) | | 1.44  (0.97, 2.14) | 0.85  (0.48, 1.51) | | |
| 29-30 | 23/370  (6.2) | | 10/87  (11.5) | | 1.85  (0.91, 3.74) | | 1.60  (0.63, 4.05) | | | | 80/369  (21.7) | | | 19/87  (21.8) | | 1.01  (0.65, 1.57) | 1.17  (0.67, 2.02) | | |
| 30-31 | 22/406  (5.4) | | 12/147  (8.2) | | 1.51  (0.77, 2.97) | | 1.16  (0.54, 2.48) | | | | 84/403  (20.8) | | | 52/145  (35.9) | | 1.72  (1.29, 2.30) | 1.50  (1.05, 2.14) | | |
| 31-32 | 22/484  (4.6) | | 8/151  (5.3) | | 1.17  (0.53, 2.56) | | 0.90  (0.37, 2.19) | | | | 99/480  (20.6) | | | 34/150  (22.7) | | 1.10  (0.78, 1.55) | 0.97  (0.64, 1.47) | | |
| 32-33 | 35/571  (6.1) | | 10/177  (5.7) | | 0.92  (0.47, 1.82) | | 0.48  (0.23, 0.98) | | | | 131/566 (23.1) | | | 63/177  (35.6) | | 1.54  (1.20, 1.97) | 1.45  (1.07, 1.95) | | |
| 33-34 | 25/619  (4.0) | | 8/194  (4.1) | | 1.02  (0.47, 2.23) | | 0.45  (0.20, 1.04) | | | | 152/615 (24.7) | | | 57/193  (29.5) | | 1.19  (0.92, 1.55) | 0.92  (0.66, 1.28) | | |
| 34-35 | 23/744  (3.1) | | 8/254  (3.2) | | 1.02  (0.46, 2.25) | | 1.01  (0.42, 2.45) | | | | 190/741 (25.6) | | | 66/254  (26.0) | | 1.01  (0.80, 1.29) | 0.90  (0.66, 1.23) | | |
| 35-36 | 28/875  (3.2) | | 4/103  (1.3) | | 0.36  (0.05, 2.81) | | 0.19  (0.09, 1.49) | | | | 189/869 (21.8) | | | 80/303  (26.4) | | 1.48  (0.94, 2.32) | 1.19  (0.69, 2.05) | | |
| **Pooled^c^** |  | |  | |  | | **0.92**  **(0.68, 1.19)** | | | |  | | |  | |  | **1.09**  **(0.97, 1.23)** | | |
|  | **Outcome: VSGA** | | | | | | | | | **Outcome: Preterm birth** | | | | | | | | |  |
| 24-25 | 16/124  (20.2) | | 9/51  (17.7) | | 1.37  (0.65, 2.89) | | 1.17  (0.44, 3.08) | | | | 42/124  (33.9) | | | 23/52  (44.2) | | 1.31  (0.88, 1.93) | 1.67  (0.84, 3.29) | | |
| 25-26 | 24/175  (13.7) | | 6/59  (10.2) | | 0.74  (0.32, 1.73) | | 0.62  (0.24, 1.58) | | | | 44/176  (25.0) | | | 27/60  (45.0) | | 1.80  (1.23, 2.63) | 1.78  (1.16, 2.74) | | |
| 26-27 | 22/210  (10.5) | | 16/82  (19.5) | | 1.86  (1.03, 3.36) | | 2.14  (1.07, 4.27) | | | | 47/212  (22.2) | | | 36/83  (43.4) | | 1.96  (1.38, 2.78) | 1.37  (0.89, 2.10) | | |
| 27-28 | 33/313  (10.5) | | 16/80  (20.0) | | 1.90  (1.10, 3.27) | | 1.04  (0.48, 2.28) | | | | 75 /317  (23.7) | | | 36/82  (43.9) | | 1.86  (1.35, 2.54) | 1.94  (1.13, 3.33) | | |
| 28-29 | 25/310  (8.1) | | 18/84  (21.4) | | 2.66  (1.52, 4.63) | | 1.70  (0.83, 3.50) | | | | 95 /313  (30.4) | | | 38/84  (45.2) | | 1.49  (1.12, 1.99) | 1.00  (0.61, 1.65) | | |
| 29-30 | 34/369  (9.2) | | 9/87  (10.3) | | 1.12  (0.56, 2.25) | | 0.72  (0.27, 1.90) | | | | 115/370  (31.1) | | | 38/87  (43.7) | | 1.41  (1.06, 1.86) | 1.26  (0.86, 1.84) | | |
| 30-31 | 39/403  (9.7) | | 31/145  (21.4) | | 2.21  (1.43, 3.40) | | 1.73  (1.02, 2.92) | | | | 125/406  (30.8) | | | 65/147  (44.2) | | 1.44  (1.14, 1.81) | 0.95  (0.70, 1.30) | | |
| 31-32 | 44/480  (9.2) | | 16/150  (10.7) | | 1.16  (0.68, 2.00) | | 1.09  (0.59, 2.01) | | | | 132/484 (27.3) | | | 73/151  (48.3) | | 1.77  (1.42, 2.21) | 1.25  (0.94, 1.67) | | |
| 32-33 | 63/566  (11.1) | | 31/177  (17.5) | | 1.57  (1.06, 2.34) | | 1.21  (0.76, 1.93) | | | | 154/571 (27.0) | | | 72/177  (40.7) | | 1.51  (1.21, 1.89) | 1.01  (0.76, 1.35) | | |
| 33-34 | 79/615  (12.8) | | 33/193  (17.1) | | 1.33  (0.92, 1.93) | | 0.98  (0.62, 1.54) | | | | 163/619 (26.3) | | | 73/194  (37.6) | | 1.43  (1.14, 1.79) | 0.96  (0.71, 1.31) | | |
| 34-35 | 87/741  (11.7) | | 29/254  (11.4) | | 0.97  (0.65, 1.44) | | 0.62  (0.40, 0.97) | | | | 178/744 (23.9) | | | 70/254  (27.6) | | 1.15  (0.91, 1.46) | 0.75  (0.56, 1.00) | | |
| 35-36 | 88/869  (10.1) | | 35/303  (11.6) | | 1.14  (0.79, 1.65) | | 0.87  (0.58, 1.30) | | | | 167/875 (19.1) | | | 55/305  (18.0) | | 0.94  (0.72, 1.24) | 0.81  (0.57, 1.16) | | |
| **Pooled^c^** |  | |  | |  | | **1.05**  **(0.88, 1.25)** | | | |  | | |  | |  | **1.09**  **(0.96, 1.22)** | | |
|  | **Outcome: Very preterm birth** | | | | | | | | | | | **Outcome: Neonatal death** | | | | | | |  |
| 24-25 | 23/124  (18.6) | | 18/52  (34.6) | | 1.87  (1.10, 3.15) | | 2.07  (0.94, 4.53) | | | | 4/107  (3.7) | | | 4/39  (10.3) | | 2.74  (0.72, 10.44) | 2.54  (0.55, 11.78) | | |
| 25-26 | 23/176  (13.1) | | 9/60  (15.0) | | 1.15  (0.56, 2.34) | | 1.19  (0.48, 2.93) | | | | 5/162  (3.1) | | | 4/50  (8.0) | | 2.59  (0.72, 9.29) | 1.65  (0.37, 7.37) | | |
| 26-27 | 24/212  (11.3) | | 18/83  (21.7) | | 1.92  (1.10, 3.34) | | 0.93  (0.49, 1.77) | | | | 8/199  (4.0) | | | 5/72  (6.9) | | 1.73  (0.58, 5.11) | 1.08  (0.33, 3.56) | | |
| 27-28 | 31/317  (9.8) | | 16/82  (19.5) | | 2.00  (1.15, 3.47) | | 0.91  (0.37, 2.25) | | | | 10/295  (3.4) | | | 4/73  (5.5) | | 1.62  (0.52, 5.01) | 0.93  (0.22, 3.93) | | |
| 28-29 | 31/313  (9.9) | | 17/84  (20.2) | | 2.04  (1.19, 3.51) | | 0.67  (0.33, 1.36) | | | | 8/289  (2.8) | | | 2/73  (2.7) | | 0.99  (0.21, 4.56) | 1.07  (0.17, 6.94) | | |
| 29-30 | 27/370  (7.3) | | 11/87  (12.64) | | 1.73  (0.89, 3.36) | | 1.49  (0.57, 3.90) | | | | 6/347  (1.7) | | | 0/77  (0.0) | | -- | -- | | |
| 30-31 | 22 /406  (5.4) | | 8/147  (5.4) | | 1.00  (0.46, 2.21) | | 0.56  (0.24, 1.32) | | | | 9/384  (2.3) | | | 5/135  (3.7) | | 1.58  (0.54, 4.63) | 1.67  (0.51, 5.50) | | |
| 31-32 |  | |  | | -- | | -- | | | | 6/461  (1.3) | | | 3/143  (2.1) | | 1.61  (0.41, 6.36) | 1.29  (0.30, 5.45) | | |
| 32-33 |  | |  | | -- | | -- | | | | 8/534  (1.5) | | | 1/167  (0.6) | | 0.40  (0.05, 3.17) | 0.19  (0.02, 1.53) | | |
| 33-34 |  | |  | | -- | | -- | | | | 6/593  (1.0) | | | 2/168  (1.1) | | 1.06  (0.22, 5.22) | 0.60  (0.12, 3.00) | | |
| 34-35 |  | |  | | -- | | -- | | | | 6/721  (0.8) | | | 4/244  (1.6) | | 1.97  (0.56, 6.92) | 1.38  (0.37, 5.16) | | |
| 35-36 |  | |  | | -- | | -- | | | | 5/846  (0.6) | | | 5/300  (1.7) | | 2.82  (0.82, 9.67) | 2.71  (0.78, 9.43) | | |
| **Pooled^c^** |  | |  | |  | | **1.05**  **(0.78, 1.47)** | | | |  | | |  | |  | **1.23**  **(0.68, 2.24)** | | |
|  | **Outcome: Severe gestational hypertension** | | | | | | |  | | | | | | | | | | |  |
| 24-25 | 24/124  (19.4) | 10/52  (19.2) | | 0.99  (0.51, 1.93) | | 0.82  (0.33, 2.02) | | |  | | | |  | |  | | |  |  |
| 25-26 | 30/176  (17.0) | 15/60  (25.0) | | 1.47  (0.85, 2.53) | | 0.85  (0.44, 1.62) | | |  | | | |  | |  | | |  |  |
| 26-27 | 34/212  (16.0) | 25/83  (30.1) | | 1.88  (1.20, 2.94) | | 1.54  (0.91, 2.61) | | |  | | | |  | |  | | |  |  |
| 27-28 | 53/317  (16.7) | 16/82  (19.5) | | 1.17  (0.71, 1.93) | | 0.65  (0.30, 1.41) | | |  | | | |  | |  | | |  |  |
| 28-29 | 62/313  (19.8) | 15/84  (17.87) | | 0.90  (0.54, 1.50) | | 0.64  (0.33, 1.24) | | |  | | | |  | |  | | |  |  |
| 29-30 | 64/370  (17.3) | 12/87  (13.8) | | 0.80  (0.45, 1.41) | | 0.73  (0.38, 1.40) | | |  | | | |  | |  | | |  |  |
| 30-31 | 56/406  (13.8) | 42/147  (28.6) | | 2.07  (1.46, 2.95) | | 1.39  (0.91, 2.13) | | |  | | | |  | |  | | |  |  |
| 31-32 | 62/484  (12.8) | 27/151  (17.9) | | 1.40  (0.92, 2.11) | | 0.88  (0.54, 1.43) | | |  | | | |  | |  | | |  |  |
| 32-33 | 75/571  (13.1) | 32/177  (18.1) | | 0.19  (0.02, 1.53) | | 0.97  (0.62, 1.51) | | |  | | | |  | |  | | |  |  |
| 33-34 | 92/619  (14.9) | 27/194  (13.9) | | 0.94  (0.63, 1.39) | | 1.02  (0.55, 1.89) | | |  | | | |  | |  | | |  |  |
| 34-35 | 98/744  (13.2) | 40/254  (15.8) | | 1.20  (0.85, 1.68) | | 0.93  (0.63, 1.36) | | |  | | | |  | |  | | |  |  |
| 35-36 | 93/875  (10.6) | 14/305  (4.6) | | 0.43  (0.25, 0.75) | | 0.34  (0.19, 0.61) | | |  | | | |  | |  | | |  |  |
| **Pooled^c^** |  |  | |  | | **0.88**  **(0.74, 1.07)** | | |  | | | |  | |  | | |  |  |

RR: risk ratio; CI: confidence interval; aRR: adjusted risk ratio; SGA: small-for-gestational-age; VSGA: very small-for-gestational-age.

^a^ Models were adjusted using stabilized inverse probability weights accounting for maternal age, parity, history of preterm or stillbirth, occupation, HIV status, trimester of first antenatal care, first trimester weight, first SBP and DBP at non-severe gestational hypertension diagnosis, SBP and DBP at start of trial, delivery site, and calendar year of delivery.

^b^ 95% CI for the pooled models were calculated via bootstrapping with 200 samples.

^c^ The adjusted pooled model additionally includes “trial” as an adjustment variable.

# Table Sx2. Risk ratios from subgroup analyses by HIV status, first trimester weight, and parity on stillbirth and SGA by antihypertensive initiation between 24- and 35-weeks’ gestation, the Tsepamo Study (2014-2022).

|  | **Overall** | **HIV status** | | **First trimester weight** | | **Parity** | |
| --- | --- | --- | --- | --- | --- | --- | --- |
|  |  | **Living with HIV** | **Not living with HIV** | **<50 kg** | **≥80 kg** | **Nulliparous** | **Primiparous** |
|  | **Outcome: Stillbirth** | | | | | | |
| No events / Total no person-trials (%) Non-initiators | 269/5211 (5.2) | 93/1094 (8.5) | 176/4117 (4.3) | 82/1572 (5.2) | 200/5036 (4.0) | 95/2152 (4.4) | 174/3059 (5.70) |
| No events / Total no person-trials (%) Initiators | 111/1474 (7.5) | 35/328 (10.7) | 76/1347 (5.6) | 20/249  (8.0) | 36/854  (4.2) | 42/672  (6.2) | 69/1003 (6.9) |
| Pooled aRR^a^  (95% CI)^b^ | 0.92  (0.68, 1.19) | 0.64  (0.43, 1.03) | 0.92  (0.59, 1.32) | 0.54  (0.28, 0.97) | 0.65  (0.42, 1.01) | 0.98  (0.62, 1.54) | 0.88  (0.65, 1.18) |
|  | **Outcome: SGA** | | | | | | |
| No events / Total no person-trials (%) Non-initiators | 1141/5175 (22.0) | 352/1092 (32.2) | 789/4083 (19.3) | 476/1565 (30.4) | 682/5002 (13.6) | 470/2138 (22.0) | 671/3037 (22.1) |
| No events / Total no person-trials (%) Initiators | 467/1665 (28.0) | 120/326 (36.8) | 347/1339 (25.9) | 113/248 (45.6) | 147/849 (17.3) | 192/669 (28.7) | 275/996 (27.6) |
| Pooled aRR^a^  (95% CI)^b^ | 1.09  (0.97, 1.23) | 1.06  (0.85, 1.39) | 1.12  (0.97, 1.29) | 1.57  (1.10, 2.02) | 1.12  (0.90, 1.47) | 1.03  (0.82, 1.26) | 1.12  (0.93, 1.32) |
|  | **Outcome: VSGA** | | | | | | |
| No events / Total no person-trials (%) Non-initiators | 554/5175 (10.7) | 166/1092 (15.2) | 388/4083 (9.5) | 233/1565 (14.9) | 296/5002 (5.9) | 213/2138 (10.0) | 341/3037 (11.2) |
| No events / Total no person-trials (%) Initiators | 249/1665 (15.0) | 54/326 (16.6) | 195/1339 (14.6) | 67/248 (27.0) | 66/849  (7.8) | 108/669 (16.1) | 141/996 (14.2) |
| Pooled aRR^a^  (95% CI)^b^ | 1.05  (0.88, 1.25) | 0.85  (0.58, 1.27) | 1.19  (0.98, 1.44) | 1.16  (0.71, 1.71) | 1.15  (0.79, 1.62) | 1.10  (0.79, 1.48) | 1.01  (0.81, 1.27) |
|  | **Outcome: Preterm birth** | | | | | | |
| No events / Total no person-trials (%) Non-initiators | 1337/5211 (25.7) | 352/1094 (32.2) | 985/4117 (23.9) | 591/1572 (37.6) | 850/5036 (16.9) | 558/2152 (25.9) | 779/3059 (25.5) |
| No events / Total no person-trials (%) Initiators | 606/1474 (41.1) | 135/328 (41.2) | 471/1348 (34.9) | 119/249 (47.8) | 226/854 (26.5) | 254/672 (37.8) | 352/1004 (35.1) |
| Pooled aRR^a^  (95% CI)^b^ | 0.92  (0.68, 1.19) | 1.04  (0.76, 1.37) | 1.11  (0.94, 1.28) | 0.74  (0.54, 1.07) | 1.12  (0.90, 1.39) | 1.07  (0.87, 1.32) | 1.03  (0.92, 1.17) |
|  | **Outcome: Very preterm birth** | | | | | | |
| No events / Total no person-trials (%) Non-initiators | 181/1918 (9.4) | 43/394 (10.9) | 138/1524 (9.1) | 69/545 (12.66) | 114/2409 (4.7) | 78/774 (10.1) | 103/1144 (9.0) |
| No events / Total no person-trials (%) Initiators | 97/595 (16.3) | 23/126 (18.3) | 74/469 (15.8) | 17/89 (19.10) | 38/352 (10.8) | 36/215 (16.7) | 61/380 (16.0) |
| Pooled aRR^a^  (95% CI)^b^ | 1.05  (0.78, 1.47) | 0.83  (0.45, 1.72) | 1.18  (0.75, 1.86) | 0.45  (0.21, 1.31) | 1.08  (0.69, 1.67) | 0.99  (0.58, 1.98) | 1.02  (0.65, 1.47) |
|  | **Outcome: Neonatal death** | | | | | | |
| No events / Total no person-trials (%) Non-initiators | 81/4938 (1.6) | 20/1000 (2.0) | 61/3938 (1.6) | 11/1492 (0.7) | 68/4828 (1.4) | 24/2056 (1.2) | 57/2883 (2.0) |
| No events / Total no person-trials (%) Initiators | 39/1541 (2.5) | 8/292  (2.7) | 31/1267 (2.4) | 6/229  (2.6) | 17/815  (2.1) | 12/629  (1.9) | 27/930  (2.9) |
| Pooled aRR^a^  (95% CI)^b^ | 1.23  (0.68, 2.24) | 1.16  (0.34, 2.76) | 1.29  (0.79, 2.11) | 1.12  (0.36, 5.50) | 1.17  (0.48, 2.00) | 1.68 (0.49, 5.77) | 1.30  (0.67, 2.30) |
|  | **Outcome: Severe gestational hypertension** | | | | | | |
| No events / Total no person-trials (%) Non-initiators | 743/5211 (14.2) | 150/1094 (13.7) | 593/4117 (14.4) | 130/1572 (8.3) | 681/5036 (13.5) | 253/2152 (11.8) | 490/3059 (16.0) |
| No events / Total no person-trials (%) Initiators | 275/1676 (16.4) | 52/328 (15.8) | 223/1348 (16.5) | 38/249 (15.3) | 123/854 (14.4) | 96/672 (14.3) | 179/1004 (35.1) |
| Pooled aRR^a^  (95% CI)^b^ | 0.88  (0.74, 1.07) | 0.92  (0.49, 1.64) | 0.92  (0.74, 1.16) | 0.67  (0.39, 1.15) | 0.86  (0.68, 1.10) | 0.93  (0.70, 1.25) | 0.94  (0.77, 1.29) |

^a^ Models were adjusted using stabilized inverse probability weights accounting for maternal age, parity, history of preterm or stillbirth, occupation, HIV status, trimester of first antenatal care, first trimester weight, first SBP and DBP at non-severe gestational hypertension diagnosis, SBP and DBP at start of trial, delivery site, and calendar year of delivery. The adjusted pooled model additionally includes “trial” as an adjustment variable.

^b^ 95% CI for the pooled models were calculated via bootstrapping with 200 samples.
